# When Success Is Surprising: Children’s Ability to Use Surprise to Infer Competence

**DOI:** 10.1162/opmi.a.2

**Published:** 2025-07-07

**Authors:** Mika Asaba, Yang Wu, Brandon Carrillo, Hyowon Gweon

**Affiliations:** Department of Psychology, Yale University, New Haven, CT, USA; Department of Psychology, University of Toronto Scarborough, Toronto, ON, Canada; Department of Psychology, Stanford University, Stanford, CA, USA

**Keywords:** social cognition, affective cognition, cognitive development, theory of mind, academic achievement

## Abstract

How do we learn who is good at what? Building on the idea that humans draw rich inferences from others’ emotional expressions, here we ask whether others’ surprised reactions to performance outcomes can elicit inferences about competence. Across three experiments, participants were asked to choose “who is better” in scenarios where two students performed identically on the same task but their teacher expressed surprise to only one of them. In Experiment 1 (*n* = 60, adults) and Experiment 2 (*n* = 90, 6- to 8-year-old children), participants’ responses were modulated by not only the students’ performance outcomes (success or failure) but also the teacher’s response to the outcomes (surprise or no surprise). Specifically, participants preferentially chose the student who did not elicit the teacher’s surprise as more competent when both students succeeded, but chose the student who elicited surprise when both failed. Experiment 3a (*n* = 150, 4- to 8-year-olds) replicated this pattern in 6- to 8-year-olds as a group—but not in 4- to 5-year-olds—with increasing robustness with age. Finally, this pattern was significantly reduced in Experiment 3b where the teacher’s surprise was directed at an irrelevant event rather than the student’s performance (*n* = 90, 6- to 8-year-olds). Taken together, these results suggest that even non-valenced emotional reactions to performance outcomes—being surprised at someone’s success or failure—can inform inferences about valenced qualities such as competence. More broadly, the current findings demonstrate that emotional expressions we observe in our daily lives can lead to nuanced yet consequential social judgments.

## INTRODUCTION

Imagine two students in a classroom. John solves a very difficult math problem, and his teacher looks surprised by his success. Noah solves the same problem, but his teacher shows no surprise at all. Who is better at math, John or Noah? You might intuitively judge that Noah is better than John, even though the two students achieved the same outcome. How is this possible? This example highlights how emotional reactions to performance outcomes can shape our inferences about who is good at what. Although emotional cues are pervasive in young children’s lives, research on their role in reasoning about competence is rather limited. The current study aims to address this gap by exploring how children use emotional reactions, particularly surprise, to make judgments about competence.

Reasoning about competence is critical to our everyday decisions and social interactions, such as whom to help (Sierksma & Shutts, 2021), whom to ask for help (Sobel & Kushnir, 2013), and how to allocate our own and others’ efforts (Baer & Odic, 2022; Magid et al., 2018; Magid & Schulz, 2015; Xiang et al., 2024). Evaluating someone’s competence, however, is not a trivial task; it is an abstract, internal quality of people that cannot be directly observed and must be inferred from observable data.

One important source of information for reasoning about competence comes from how someone performed on a task and the nature of the task itself. Beyond using simple performance outcomes such as successes and failures (Cimpian et al., 2017), prior work suggests that even young children judge others’ competence by considering the difficulty of the task (Heyman et al., 2003; Leonard et al., 2019; Muradoglu & Cimpian, 2020). For example, preschool-aged children readily understand that an agent who completed a difficult task (e.g., building a tall block tower) is better at the task than an agent who completed a relatively easier task in the same amount of time (Leonard et al., 2019). Thus, even young children can use clear differences in performance outcomes or task difficulty to reason about who is more competent at a task.

Another source of information about competence is social feedback, such as a teacher’s evaluation of a student’s performance. Prior work suggests that the content of evaluative feedback can also elicit nuanced inferences about competence (Cimpian et al., 2007; Henderlong & Lepper, 2002; Mueller & Dweck, 1998). For example, elementary school-aged children who received praise directed at their intelligence (“You must be smart”) were more likely to attribute their subsequent failures to a lack of ability than students who received praise directed at effort (“You must have worked hard”; Mueller & Dweck, 1998). Furthermore, faced with a choice between a student who was praised for low effort (“that kid never even had to work hard”) and a student who was praised for high effort (“that kid tried and tried”), even 5-year-old children judged the former to be smarter (Zhao et al., 2022). Together, these results suggest that children can use the contents of verbal feedback to reason about competence.

The current work focuses on another source of information that is common in everyday social interactions: *emotional reactions* to performance outcomes. As illustrated by the running example above, seeing how a teacher reacts to her students’ performance—being surprised by success, for instance—can elicit powerful inferences about competence. We argue that this apparent inferential leap is supported by an intuitive understanding of how one’s observations and mental states (e.g., the teacher’s prior beliefs about the students’ competence and her observation of the students’ performance) give rise to emotion (e.g., Doan et al., 2025; Harris et al., 1989; Houlihan et al., 2022; Lagattuta, 2014; Ong et al., 2019; Wellman & Liu, 2004; Wu et al., 2018). From this perspective, the teacher’s surprise at John’s (but not Noah’s) success indicates that she found John’s success more unexpected than Noah’s; assuming the teacher’s expectations are valid, this supports the inference that Noah is better than John.^1^ The current work aims to explore the development of this inferential leap from someone’s surprised reactions to another person’s competence.

Past work suggests that humans, starting early in life, use others’ emotion as a source of information for learning (for a review, see Wu et al., 2021). By 12 months, infants can use others’ emotional reactions (e.g., “wow!” or “hahaha!”) to infer the likely cause of those emotions (e.g., something exciting or funny) and use it to guide their exploration (Clément & Dukes, 2017; Moll et al., 2006, 2007; Walle et al., 2017; Wu & Gweon, 2021; Wu et al., 2017). With age, children can also use emotional cues to reason about others’ internal mental states, such as beliefs and desires (O’Brien et al., 2011; Repacholi & Gopnik, 1997; Wellman & Banerjee, 1991; Wu & Schulz, 2018). For instance, when the valence of someone’s emotional expression changes after knowing what is inside a box (e.g., from smiling to frowning), children around 5 years of age can infer that the person had a false belief about the content of the box (Wu & Schulz, 2018).

Yet, only a handful of studies thus far have investigated how children use emotion to reason about competence. In one study, presented with a scenario in which a teacher reacted to a student’s failure with pity, children under 9 years of age struggled to infer that the student has low ability (Weiner et al., 1982). It is possible that this task underestimated children’s abilities, as it used complex emotion words (e.g., pity) that younger children may not yet understand. Indeed, other studies have shown that younger school-aged children tend to evaluate individuals who receive positive nonverbal responses (e.g., smiling, nodding) as smarter, nicer, and stronger than those who receive negative nonverbal responses (e.g., frowning, headshaking; Brey & Pauker, 2019; Brey & Shutts, 2018). Critically however, these studies used clearly valenced emotional cues (e.g., smiling) that could be associated with similarly valenced traits (e.g., competence). Therefore, these studies fall short of showing that children’s judgments are grounded in their theory-like understanding of emotion.

The current study focuses on children’s reasoning about competence based on a non-valenced emotion: surprise. Unlike valenced emotional cues, which can easily be mapped onto the valence of abstract qualities, surprise is inherently neutral; it indicates that the observed event violated the emoter’s expectations. Thus, reasoning based on surprise requires the ability to use the emotion to infer what the emoter expected to happen. Previous research has demonstrated that an understanding of surprise emerges in early childhood. As early as 12 months, infants can use others’ surprise as an indicator of prediction error, anticipating an improbable statistical outcome after witnessing an experimenter’s surprise (Wu et al., 2024). Throughout early childhood, children also become increasingly capable of connecting others’ surprise with their internal mental states (e.g., ignorance, false beliefs) about the physical states of the world (Hadwin & Perner, 1991; MacLaren & Olson, 1993; Moll et al., 2006; Wellman & Banerjee, 1991; Wu & Gweon, 2021). These findings raise the possibility that the ability to use others’ surprise to infer competence may also emerge early in childhood.

We report three preregistered experiments that aim to test this possibility. The experimental scenarios were situated in a classroom context, in order to capitalize on the well-established expectation that teachers are knowledgeable and trustworthy (Gweon, 2021). In each experiment, participants were presented with vignettes in which two students achieved the same performance outcomes (both succeeded or failed) on the same task (a physical or academic activity) as their teacher observed. The teacher displayed surprise to one of them and no surprise to the other. Participants were asked to evaluate the students’ relative competence.

Our key hypothesis was that participants’ inferences about the students’ relative competence would be modulated by not just the performance outcomes (i.e., whether the students succeeded or failed) but also the teacher’s reaction to these outcomes (i.e., whether or not the teacher was surprised). Specifically, we predicted that when both students succeeded (Success condition), they would infer that the student who elicited the teacher’s surprise is less competent, whereas when both students failed (Fail condition), participants would infer that the student who elicited surprise is more competent. Because the test question was always “Who is better?”, we predicted that participants in the Success condition would choose the student who did not receive surprise, whereas those in the Fail condition would choose the student who did receive surprise.

We first show this pattern in adults (Experiment 1, *n* = 60) and 6- to 8 year-old children (Experiment 2, *n* = 90). We then further demonstrate that this ability undergoes significant developmental change between 4 to 8 years of age (Experiment 3a, *n* = 150), and address alternative accounts of our findings (Experiment 3b, *n* = 90). Preregistrations, materials and experimenter scripts, data, and analyses are here: https://osf.io/4ezvt/.

## EXPERIMENT 1

Before testing children, we first validated our hypothesis with adults. We asked whether adults would use the presence or absence of a teacher’s surprised expression to students’ performance outcomes to evaluate the students’ relative competence.

### Methods

#### Participants.

We recruited 60 adults (*M*_*Age*_(*SD*) = 32.2(13.6), Range = 18–74 yrs) via *Prolific*. All participants were in the U.S. and passed our preregistered exclusion criteria of failing to accurately answer 50% or more of the 8 check questions. Participants reported their gender as man (*n* = 30), woman (*n* = 28), or non-binary (*n* = 2), and reported their race as White (*n* = 42), Asian (*n* = 8), Black (*n* = 3), Native Hawaiian/other Pacific Islander (*n* = 1), multi-racial (*n* = 2), or other (*n* = 4).

#### Stimuli.

Stimuli were presented on an online survey platform (*Qualtrics*) as simple vignettes that depicted two students engaging in one of four activities (kicking, throwing, spelling, math) and the teacher’s reaction to their performance. Students in the vignettes were same-gendered pairs of generic cartoon characters without facial features, and their successes and failures on a given activity were marked with a green checkmark and a red “X”, respectively. The teacher’s emotional expressions to the students’ failures or successes were presented by two photos of an adult woman: One with surprise (a prototypical surprised expression), and one with no surprise (a mildly positive expression, same as the baseline expression^2^).

#### Design and Procedure.

Participants first read about a teacher and her students, who “do a bunch of different activities throughout the day”. To make it more plausible that the students’ performance might violate the teacher’s expectations, participants were told that “Sometimes the kids get lucky and do really well in these activities, and sometimes they accidentally make mistakes”, and that the teacher would look “surprised” or “not surprised”. Participants then underwent two warm-up questions that checked their understanding of the teacher’s expressions (“Is the teacher surprised or not surprised here?”) before the four test trials began.

In each trial, participants were introduced to an activity that the students were about to do: “throw the ball into the bucket” (throwing), “kick the ball into the goal” (kicking), “spell some words” (spelling), or “solve some math problems” (math). They were also told that “The teacher is watching them play and sees how they do”, and were shown the teacher’s non-surprised, baseline expression. Then, participants were shown two students attempting the activity where both students either succeeded (Success condition) or failed (Fail condition). Then, the teacher’s reaction to their performance was revealed (surprise or no surprise). To avoid any ambiguity, the images of the teacher’s expressions were clearly labeled for participants: “The teacher was *surprised* that John got the math problem right, and was *not surprised* that Noah got the math problem right.” As the two students were the same gender and had achieved the same outcomes, the key difference was whether the teacher expressed surprise (henceforth “surprise student”) or did not express surprise (“no-surprise student”) at their outcome. Participants then answered two check questions about the teacher’s emotional response to the students’ performance, one for each student: “Just to check, was the teacher surprised or not surprised that [John/Noah] got the math problem right?” With images of the students’ outcomes and the teacher’s expressions visible, participants were asked the test question: “One of these kids is better at math and the other is worse. Who is better, John or Noah?” Trials in the Fail condition were identical except that both students failed at the activity.

All participants completed four trials across two conditions: two trials in the Success condition and two trials in the Fail condition (repeated measures, within-subjects design). Each trial showed a unique activity (throwing, kicking, spelling, math). Trials were blocked by condition (i.e., Success condition, Fail condition), and condition order (Success condition first, or Fail condition first), activity order, and student gender for each activity (which activities boys versus girls did) were counterbalanced across participants.^3^ See Qualtrics survey here: https://tinyurl.com/2rbkew9x.

### Results and Discussion

**Figure 1.**
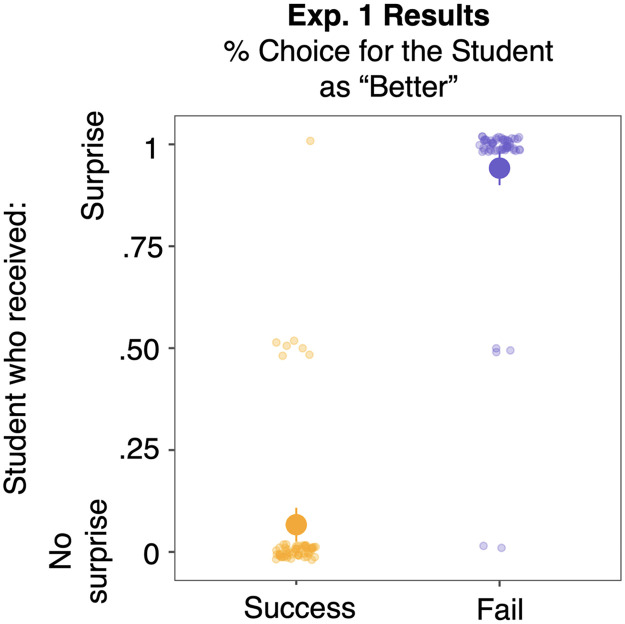
Adults’ choice of students in response to the test question “who is better” in the Success and Fail conditions (Experiment 1). Large dots show group means for the Success (yellow) and Fail (purple) conditions. Small dots show individual participants’ average responses. Error bars represent 95% confidence intervals.

Our primary question was whether adults would use the presence or absence of the teacher’s surprised expression to the students’ performance outcomes to evaluate the students’ relative competence. Following our preregistered analyses, we ran a generalized linear mixed-effects model with Condition (Success vs. Fail) as a fixed effect predicting participants’ choice of student (the surprise student or the no-surprise student), and random intercepts for Activity and random intercepts and slopes for Participants by Condition. After pruning the random effects due to singular-fit issues, our final model was: glmer(Choice ∼ Condition + (1|Activity) + (1|Participant), family = “binomial“). We found a significant effect of Condition (Success vs. Fail; *β* = −9.439, *z* = −4.317, *p* < .001), suggesting that participants’ choice of students differed across conditions. See Figure 1.

Follow-up analyses showed that adults chose the no-surprise student significantly above chance in the Success condition (93.3%, *Z* = 7.08, *p* < .001) and the surprise student significantly above chance in the Fail condition (94.2%, *Z* = 7.02, *p* < .001; Exact Wilcoxon-Pratt Signed-Rank Test). We fully replicated these findings in a separate experiment (*n* = 67 adults; see Supplementary Online Materials (SOM) Section 1).

These results provide clear evidence that adults can use surprised reactions to reason about competence. Rather than simply relying on students’ outcomes alone as indicators of competence, participants integrated the teacher’s emotional response to infer the students’ underlying competence.

## EXPERIMENT 2

Next, we tested whether early school-aged children (6- to 8-year-olds) can use surprise to draw similar inferences about competence. We start with this age range for a few reasons. First, by ages 5 and 6, children begin to use others’ surprise to infer their expectations or beliefs about concrete objects, such as the content of a box (e.g., Hadwin & Perner, 1991; MacLaren & Olson, 1993; Wellman & Banerjee, 1991). Here, as our focus is on reasoning about competence—an abstract quality of agents—we anticipated a slightly later development in using others’ surprise to infer their expectations or beliefs about competence. Second, while prior work suggests that children struggle with relative judgments of competence even in late childhood (Nicholls, 1978), more recent work has shown some success in early school-aged children, when provided with cues that clearly signal underlying competence (e.g., faster speed or verbal mentions of success; Leonard et al., 2019; Muradoglu & Cimpian, 2020). Last, children in our sample typically begin formal schooling at age six; such experience may facilitate performance-related social comparisons (Magid & Schulz, 2015; Ruble et al., 1980). As such, we focused on children 6 to 8 years of age in this experiment.

### Methods

#### Participants.

Ninety 6- to 8-year-old children (30 in each age group; *M*_*Age*_(*SD*) = 7.5(.89), Range = 6.0–8.9 yrs) were recruited online (participant database, Facebook advertisements) for a study session on Zoom. This sample size was based on a power analysis from our pilot experiment (see SOM Section 2.3), which suggested that we need 30 participants to detect a Cohen’s *d* of approximately .6 with 90% power; we collected 30 participants per age group, for 90 total, in order to run analyses within each age group. One additional child was tested but excluded due to having audio problems during the testing session (preregistered exclusion). All children passed other preregistered exclusion criteria, including failing to respond correctly to more than half of the 8 check questions during the test trials (only 7 participants missed 1 or 2 check questions). Parents reported their child’s gender as boy (*n* = 42) or girl (*n* = 48), and reported their race/ethnicity as White (*n* = 50), Asian (*n* = 15), Hispanic or Latine (*n* = 1), multi-racial (*n* = 12), other (*n* = 3) or did not specify (*n* = 9).

#### Stimuli.

The same images as in Experiment 1 were presented via a Keynote presentation controlled by the experimenter. This format allowed for minor modifications (e.g., animation and sound effects) to make the task more engaging for children. See Figure S1.

#### Design and Procedure.

Participants were tested virtually in a Zoom video call; all children viewed the experiment on a laptop, monitor, or tablet at home. The experimenter first went through an extensive set-up procedure with parents and children to ensure consistency across participants (e.g., Zoom was in full screen mode, experimenter’s video was in the same place, child could not see their own video, see Chuey et al., 2021).

**Figure 2.**
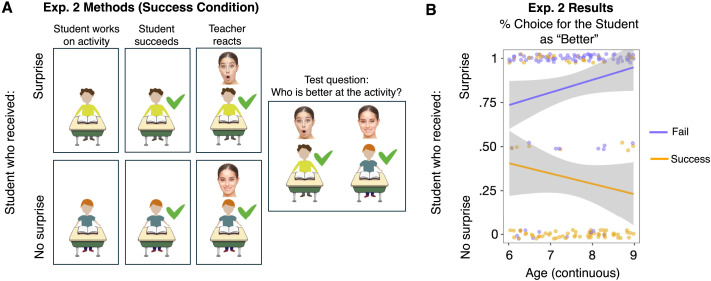
(A) Schematic of a trial in Experiment 2 (Success condition, math problem). Participants saw two students, one at a time, each of whom succeeded on the same activity (e.g., solving a math problem) and received either surprise or no surprise from their teacher. At test, participants were asked which student is better at the activity. The Fail condition was identical, except both students failed at the task. (B) Experiment 2 results, showing participants’ responses to the test question in the Success (orange) and Fail (purple) conditions. Each dot represents an individual participant’s average response. Lines show smoothed linear trends for each condition, and dark gray error bands represent 95% confidence intervals.

The experimental procedure was similar to Experiment 1, but a few changes were made to adapt the task for Zoom and make it suitable for children. First, rather than participants reading text, the experimenter narrated the task and verbally asked participants the warm-up, check, and test questions. Second, after participants labeled the teacher’s expressions for the warm-up questions and the check questions during the test trials, the experimenter either affirmed (e.g., “Yes that’s right, she’s surprised!”) or corrected (“Actually, she’s surprised!”) their answer. Third, the story was paced for children, with animations throughout to aid comprehension. Specifically, in the test trials, while adults in Experiment 1 viewed a single image with both students’ outcomes and the teachers’ expressions, children in Experiment 2 viewed one student at a time, and with animations that highlighted each student’s outcome and the teacher’s reaction. Specifically, on the left side of the screen, we first showed a student attempting the task, revealed whether they succeeded or failed, and then showed the teacher’s emotional reaction (e.g., surprise) to the outcome. Then, everything on the left side disappeared, and we showed another student on the right side with the same sequence of events, but with a different emotional reaction from the teacher (e.g., no surprise) to the outcome. Finally, for the test question, both students reappeared on the screen in their original positions, with their performance outcomes indicated with check marks or crosses, and the teacher’s expressions. Participants were explicitly reminded of the teacher’s expressions and the outcomes again, e.g.: “The teacher was *surprised* that John got the math problem right, and was *not surprised* that Noah got the math problem right” followed by our test question: “One of these kids is better at math and the other is worse. Who is better—John or Noah?” See Figure 2.

The experimental design was identical to Experiment 1. Participants underwent two trials in the Success condition and two trials in the Fail condition (trials blocked by condition), and each trial showed a unique activity (throwing, kicking, math, and spelling). Condition and activity order were counterbalanced.

### Results and Discussion

As in Experiment 1, our key question was whether participants would use the presence or absence of the teacher’s surprise to the students’ outcomes to infer the students’ relative competence. We ran a generalized linear mixed-effects model with Condition (Success vs. Fail) and Age (centered, continuous) as fixed effects, with an interaction term between Condition and Age, predicting participants’ choice of student; we included random intercepts for Activity (throwing, kicking, math, spelling) and random intercepts and slopes for Participants by Condition. (Note that the preregistered mixed-effects model, with Activity and Order as fixed effects, failed to converge; see SOM Section 3.2.1 for details.) We found a main effect of Condition (*β* = −17.948, *z* = −8.936, *p* < .001), suggesting that children’s choice of students differed across conditions. This model did not find evidence of an effect of Age (*β* = .663, *z* = .627, *p* = .531) or an interaction between Age and Condition (*β* = −1.129, *z* = −.785, *p* = .432). We conducted exploratory analyses to test whether the effect was robust within each age group. Using the same mixed-effects model without the Age and interaction terms, we found a significant effect of Condition across all age groups (6-year-olds: *β* = −5.065, *z* = −2.777, *p* = .005; 7 year-olds: *β* = −5.713, *z* = −4.070, *p* < .001; 8 year-olds: *β* = −3.263, *z* = −4.953, *p* < .001), meaning that all age groups chose different students between the Success and Fail conditions.

Our next preregistered analyses looked at participants’ performance in the Success and Fail conditions separately. As a group, children were more likely than chance to choose the no-surprise student as more competent in the Success condition (68.33%, *Z* = 3.622, *p* < .001; Exact Wilcoxon-Pratt Signed-Rank Test), and the surprise student in the Fail condition (84.44%, *Z* = 6.847, *p* < .001; Exact Wilcoxon-Pratt Signed-Rank Test).

Our preregistered, follow-up analyses examined whether the above pattern was present in each age group. In the Success condition, 6 year-olds’ performance was in the predicted direction but did not reach significance (61.67% chose the no-surprise student, *Z* = 1.347, *p* = .248), 7 year-olds’ performance was marginally significant (68.33%, *Z* = 2.043, *p* = .061), and 8 year-olds’ performance was significant (75%, *Z* = 2.88, *p* = .006). In the Fail condition, all age groups’ performance was significantly above chance (6-year-olds: 76.67% chose the surprise student, *Z* = 3.024, *p* = .004; 7-year-olds: 90%, *Z* = 4.536, *p* < .001; 8-year-olds: 86.67%, *Z* = 4.315, *p* < .001).

Taken together, we found that 6- to 8-year-old children considered a teacher’s emotional responses (surprise or no surprise) and students’ performance outcomes (success or fail) to evaluate the students’ relative competence. While these results are consistent with our prediction and in line with adults’ responses in Experiment 1, we also found an unexpected asymmetry in children’s choices: While children across all age groups reliably chose the surprise student in the Fail condition, only 8-year-olds reliably chose the no-surprise student in the Success condition, with an emerging trend in 6- and 7-year-olds. In the next experiment, we sought to address a few questions raised by these findings and provide more robust support for our hypothesis.

## EXPERIMENT 3

Experiment 3 consisted of two closely-matched experiments (3a and 3b) to address the following points. First, the findings in Experiment 2—both the overall pattern and the asymmetry across conditions—prompted questions about their robustness. Therefore, Experiment 3a sought to conceptually replicate Experiment 2 using a different method for data collection; instead of a moderated online testing method (i.e., Zoom video calls; Chuey et al., 2021), we conducted the experiment on Children Helping Science, an unmoderated online testing platform for developmental studies (Scott & Schulz, 2017).

Second, given that even 6-year-olds were showing a clear difference between conditions that becomes increasingly robust with age, one might wonder when such a pattern emerges in early childhood. Thus, in Experiment 3a, we extended the age range to include 4- and 5-year-old children.

Finally, findings in Experiment 2 could potentially be explained by alternative accounts that rely on mere associations between teachers’ emotional expressions and students’ performance outcomes. For example, in the Success condition, participants may have preferred the student who is associated with the teacher’s smile (i.e., no-surprise student) because smiling goes with success; likewise, in the Fail condition, participants may have avoided that student because smiling does not go with failure. This account is plausible particularly because in Experiment 2, both the teacher’s face and the two students’ performance outcomes (success or failure) were clearly visible on the screen at the time of the test question.

To address alternative low-level accounts, we made two key design choices that can help constrain the interpretation of our findings. First, in Experiments 3a and 3b, we removed the teacher’s face from the screen during the test question. If these images encourage children to simply associate the teacher’s facial expression with the students’ performance outcomes at test, removing the teacher’s face should no longer elicit the pattern of choices observed in Experiment 2. Second, across Experiments 3a and 3b, we manipulated whether the teacher’s expressions were directed at each student’s performance outcome as in the previous experiments (Experiment 3a) or at an irrelevant event (e.g., a bird flying, a pencil dropping; Experiment 3b). If children’s choices reflect genuine inferences about the students’ competence based on the teacher’s reactions to their performance outcomes, rather than an association between the emotional reactions and the students, then the expected pattern should emerge selectively in Experiment 3a where the emotional reactions were directed at the students.

### Experiment 3a

As mentioned above, Experiment 3a serves as a conceptual replication of Experiment 2, with an extended age range to identify potential developmental changes and minor adjustments to address alternative interpretations.

#### Methods.

##### Participants.

One hundred and fifty 4- to 8-year-old children (30 in each age group; *M*_*Age*_(*SD*) = 6.5(1.5), Range = 4.03–8.99 yrs) were recruited on Children Helping Science, an online, asynchronous data collection platform for children (Scott & Schulz, 2017). Parents reported their child’s gender as boy (*n* = 77) or girl (*n* = 73), and reported their race/ethnicity as White (*n* = 84), Asian (*n* = 24), Hispanic or Latine (*n* = 11), Black (*n* = 1), Middle Eastern or North African (*n* = 1), multi-racial (*n* = 24), or did not specify (*n* = 5). An additional five participants were excluded due to not completing the study; all other participants passed our preregistered exclusion criteria.

##### Stimuli.

Stimuli were nearly identical to Experiment 2 with one exception. In each trial, images of one of the following objects were shown on the screen as part of an irrelevant distractor event: a banana, an apple, a shoe, a hat, a paper plane, a kite, and a bird.

##### Design and Procedure.

**Figure 3.**
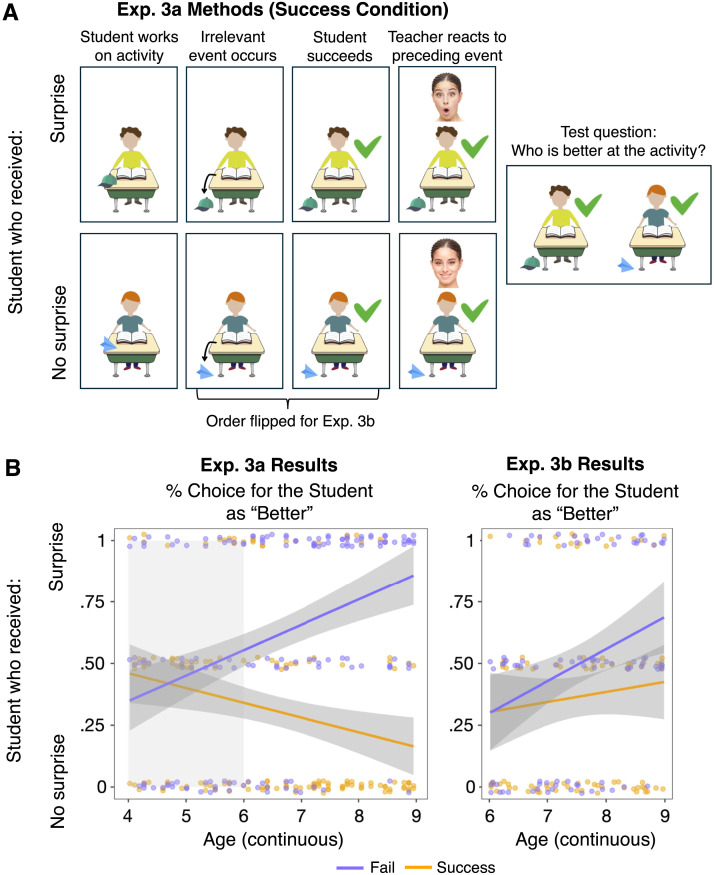
(A) Schematic of a trial in Experiment 3a (Success condition, math problem). The procedure was similar to Experiment 2, except that there was an additional irrelevant event that occurred before (Experiment 3a) or after (Experiment 3b) the student’s success. Then, the teacher showed surprise or no surprise to the preceding event, which was either the student’s success (Experiments 3a) or the irrelevant event (Experiment 3b). The Fail condition was identical, except both students failed at the task. (B) Experiment 3a and 3b results, showing participants’ responses to the test question in the Success (orange) and Fail (purple) conditions. Each dot represents an individual participant’s average response. Lines show smoothed linear trends for each condition, and dark gray error bands represent 95% confidence intervals.

As noted above, the experiment took place asynchronously without an experimenter; children clicked through the experiment on a device with a screen (e.g., a laptop or desktop computer). The procedure was similar to Experiment 2 except for the following changes. First, when introducing the story to children, we removed the phrase, “Sometimes the kids get lucky and do really well in these activities, and sometimes they accidentally make mistakes” to avoid implying that students’ performance outcomes were random rather than indicative of their competence. Second, in every test trial, an irrelevant event occurred (e.g., a bird flying, a pencil dropping) before a student attempted an activity. Then, children were told that the student is focused on the activity (e.g., “she’s focused on kicking”) to clarify that the student was not distracted by the irrelevant event. The student either succeeded or failed, followed by the teacher’s emotional response (either surprise or no surprise). The timing of these events made it clear that the teacher’s reaction was directed at the student’s performance rather than the irrelevant event. As in Experiment 2, each student was shown one at a time, with the sequence of the first student on the left side of the screen, then disappearing, followed by the sequence of the second student on the right side of the screen. Third, given that children were responding by clicking on a screen (rather than verbally on Zoom), we modified both the warm-up questions at the beginning of the experiment and the check questions in each trial. We presented both the teacher’s surprised and unsurprised expressions on the screen and asked: “Could you please click on the face where the teacher is surprised?” and “Could you please click on the face where the teacher is not surprised?” Finally, at the time of the final test question, the screen no longer showed the teachers’ faces. See Figure 3.

#### Results and Discussion.

Experiment 3a was a conceptual replication of Experiment 2 with a few changes in the stimuli and procedure. Note that these were relatively minor changes that were unlikely to affect children’s inferences about competence; thus, we expected Experiment 3a to largely replicate the pattern in Experiment 2. Critically however, if children in Experiment 2 were simply associating the outcome and the teacher’s facial expression at test, Experiment 3a would fail to replicate the earlier results.

We preregistered the logistic mixed-effects model used in Experiment 2. We included Condition (Success vs. Fail) and Age (centered, continuous) as fixed effects, with an interaction term between Condition and Age, predicting participants’ choice of student; we also included random intercepts for Activity (throwing, kicking, math, spelling) and random intercepts and slopes for Participants by Condition. This analysis revealed a main effect of Condition (*β* = −1.937, *z* = −5.773, *p* < .001) and Age (*β* = .448, *z* = 2.890, *p* = .004), and an interaction between Condition and Age (*β* = −1.076, *z* = −5.068, *p* < .001). See Figure 3.

For each age group, we ran the same mixed-effects model without the Age and interaction term (preregistered as a secondary analysis). We did not find an effect of Condition in 4-year-olds (*β* = −.283, *z* = −.727, *p* = .467) or 5-year-olds (*β* = −.007, *z* = −.017, *p* = .986). However, replicating Experiment 2, there was an effect of Condition in 6-, 7-, and 8-year-olds (6 year-olds: *β* = −1.008, *z* = −2.072, *p* = .038; 7 year-olds: *β* = −3.26, *z* = −4.65, *p* < .001; 8 year-olds: *β* = −3.524, *z* = −4.725, *p* < .001), suggesting that children’s choice of students differed across conditions.

Our next preregistered analyses looked at participants’ performance in the Success and Fail conditions separately. As a group, we found that participants selectively chose the no-surprise student in the Success condition (66.7%, *Z* = 4.811, *p* < .001; Exact Wilcoxon-Pratt Signed-Rank Test) and showed the reverse pattern in the Fail condition, choosing the surprise student (63%, *Z* = 3.806, *p* < .001; Exact Wilcoxon-Pratt Signed-Rank Test).

Then, we ran a preregistered secondary analysis examining each age group’s responses within the Success and Fail conditions separately. Consistent with the lack of effect of condition, 4- and 5-year-olds’ choice of students did not differ from chance (4-year-olds: Success, 61.29% chose the no-surprise student, *Z* = 1.807, *p* = .119; Fail, 45.16% chose the surprise student, *Z* = −.689, *p* = .648; 5-year-olds: Success, 50.0%, *Z* = 0, *p* = 1; Fail, 50%, *Z* = 0, *p* = 1). While we did find an effect of condition in 6, 7, and 8-year-old groups, 6-year-olds’ responses in each condition were not significantly above chance (6-year-olds: Success, 61.67%, *Z* = 1.53, *p* = .189; Fail, 61.67%, *Z* = 1.53, *p* = .189), but 7-year-olds and 8-year-olds showed above-chance responses (7-year-olds: Success, 81.03%, *Z* = 3.530, *p* < .001; Fail, 82.75%, *Z* = 4.146, *p* < .001; 8-year-olds: Success, 79.03%, *Z* = 3.230, *p* < .001; Fail, 82.26%, *Z* = 4.08, *p* < .001). These results suggest that the ability to use the presence or absence of surprise to infer competence becomes increasingly robust over this age range. Additionally, unlike Experiment 2 where we found an asymmetric pattern across conditions, we found similar increase in performance with age in both conditions, suggesting that the asymmetric pattern in Experiment 2 was not robust.

Finally, to facilitate comparison with Experiment 2, we ran additional exploratory analyses using data from 6- to 8-year-olds (the age range of Experiment 2). Consistent with Experiment 2, the mixed-effects model (see SOM Section 3.3.1 for full details) detected a main effect of Condition (*β* = −2.768, *z* = −7.664, *p* < .001). Unlike Experiment 2, we also detected a marginal effect of Age (*β* = .511, *z* = 1.953, *p* = .051) and an interaction between Condition and Age (*β* = −1.266, *z* = −3.757, *p* < .001). Further, as a group, 6- to 8-year-olds selectively chose the no-surprise student in the Success condition (*Z* = 5.033, *p* < .001) and the surprise student in the Fail condition (*Z* = 5.093, *p* < .001). Thus, while the age effects were more pronounced here than in Experiment 2, we largely replicated the effects of 6- to 8-year-olds as a group.

Taken together, we find evidence that 6- to 8-year-old children can reason about competence based on others’ surprised reactions to performance outcomes, and that this ability becomes more robust with age. By extending the age range to include 4- and 5-year-olds, the current results also reveal a developmental change; we find no evidence of this ability in children younger than 6. Finally, these data replicate Experiment 2 without presenting the teacher’s face at test, suggesting that the results from Experiment 2 cannot be explained by mere associations between success and smiling (i.e., the teacher’s smile) or failure and not smiling (i.e., the teacher’s surprised face).

In Experiment 3b, we sought to test whether these effects are selectively found in cases where emotional expressions provide relevant information about competence. That is, if children are genuinely drawing an inference about the student’s competence based on the teacher’s emotional reaction to their performance, these effects should disappear if the teacher’s reaction is directed at an irrelevant event.

### Experiment 3b

Experiment 3b was designed as a closely-matched control to Experiment 3a. Using the same set of events in Experiment 3a, in Experiment 3b, we changed the order of the events such that the teacher’s expression was directed at an irrelevant event, rather than the student’s performance. The purpose of this experiment was to further constrain the interpretation of our findings by testing whether children no longer show a difference across conditions when the teacher’s surprise is unrelated to the students’ performance. Thus, in Experiment 3b, we focused on the age groups that showed the expected difference between the Fail and Success conditions in previous experiments: 6- to 8-year-olds.

#### Methods.

##### Participants.

Ninety 6- to 8-year-old children (30 in each age group; *M*_*Age*_(*SD*) = 7.55(.93), Range = 6.02–8.99) were recruited on Children Helping Science. Parents reported their child’s gender as boy (*n* = 51) or girl (*n* = 43), and reported their race/ethnicity as White (*n* = 49), Asian (*n* = 16), Hispanic or Latine (*n* = 6), Black (*n* = 2), American Indian or Alaska Native (*n* = 2), multi-racial (*n* = 14), or did not specify (*n* = 1).

##### Stimuli.

Stimuli were identical to Experiment 3a.

##### Design and Procedure.

The procedure was identical to Experiment 3a, except for the order of events during the trials. Specifically, the order of events for each student in a test trial was: the student attempts the task, the performance outcome is revealed (success or failure), the irrelevant event occurs, and finally, the teacher’s emotional expression is shown (surprise or no surprise). After each student is shown, at test, both students appear back on the screen with their performance outcomes indicated with check marks or crosses. The test question was: “Both John and Noah got the same math problem right. The teacher was surprised here at the hat. The teacher was not surprised here at the paper airplane. One of these kids is better at math and the other is worse. Who is better?”

#### Results.

Our first preregistered analysis was the same logistic mixed-effects model (i.e., identical fixed-effects and random-effects structure) as in Experiment 3a. Counter to our prediction, this model did reveal an effect of Condition (*β* = 2.536, *z* = −2.811, *p* = .005) but no effect of Age (*β* = −.209, *z* = −.963, *p* = .336), and a trending interaction between Condition and Age that did not reach significance (*β* = −.470, *z* = −1.752, *p* = .080).

Next, our second preregistered analysis was to compare participants’ performance in this experiment to the 6- to 8-year-old participants from Experiment 3a: Even though we found an effect of condition in Experiment 3b, is it significantly smaller than what we observed in Experiment 3a? To answer this question, we ran a logistic mixed-effects model with Experiment, Condition, and their interaction as fixed effects, and random intercepts for activity and random intercepts and slopes for Participant by Condition. As predicted, we did find main effects of both Experiment (*β* = −1.268, *z* = −3.560, *p* < .001) and Condition (*β* = .937, *z* = 2.625, *p* = .009), and a significant interaction between the two (*β* = 2.304, *z* = 4.266, *p* < .001).

To further probe the difference between experiments, as an exploratory analysis, we asked how many individual participants in Experiment 3b showed the predicted pattern of response from Experiment 3a, where the emotional reactions were directed at students’ performance outcomes. Consistent with the significant difference between experiments, only 14.4% (13 of 90) participants in Experiment 3b showed the predicted pattern as in Experiment 3a in all 4 trials, as opposed to 45.6% of participants in Experiment 3a (41 of 90); this difference was significant (*p* < .001, Fisher’s Exact Test). See SOM Sections 3.2.2, 3.3.2, and 3.4.1 for participant-level results for all other experiments.

Collectively, we find that the overall pattern of results was diminished when the teacher’s expression was directed at an irrelevant object rather than the students’ performance. However, the effect of condition was not completely eliminated, and we discuss this point below in the General Discussion.

## GENERAL DISCUSSION

Across three preregistered experiments, we examined adults’ and children’s ability to use information about others’ emotions—specifically, the presence or absence of surprise—to infer relative competence. When two students succeeded at the same task, adults (Experiment 1) and 6- to 8-year-olds (Experiment 2) selectively chose the student whose success did not elicit the teacher’s surprise as more competent. When both students failed, they instead chose the student who did elicit the teacher’s surprise. Experiment 3a replicated this pattern in 6- to 8-year-olds as a group—but not in 4- to 5-year-olds—with increasing robustness with age. Critically, although participants chose different students between the Fail and Success conditions in Experiment 2 and 3a, this effect was significantly reduced when the teacher’s reaction was directed towards an irrelevant distractor event (Experiment 3b). Collectively, these results suggest a developing ability to use others’ surprised reactions (or the lack thereof) to performance outcomes to guide inferences about competence.

Although prior work has shown that people can use valenced emotional signals to make social evaluations (Brey & Pauker, 2019; Brey & Shutts, 2018; Skinner et al., 2017, 2020), our work found that both adults and school-aged children can use the presence or absence of a nonvalenced emotion—surprise—to make an inherently valenced judgment: who is better at a task. This indicates that, in addition to performance outcomes and others’ verbal feedback (e.g., Cimpian et al., 2017; Mueller & Dweck, 1998; Zhao et al., 2022), even valence-neutral emotional reactions like surprise can elicit inferences about competence.

Consistent with our hypothesis, children in our study were less likely to draw inferences about competence when surprise was directed at an irrelevant event (Experiment 3a vs. Experiment 3b). Rather unexpectedly, however, we also found a small but significant effect of condition. How can this be explained? First, it is worth noting that unlike other experiments, Experiment 3b had no obvious, intuitive answer; even though children did not receive any relevant evidence to infer the students’ relative competence, they still had to choose who is better. This may have led children to generate their own interpretations of the scenario and try to choose a reasonable answer. For example, although the teacher’s emotional reaction followed the appearance of an irrelevant event rather than the outcome of each student, some children may have still interpreted this reaction as relevant to the student’s outcome, in order to rationalize the situation and answer the forced-choice question. From this perspective, the small effect in Experiment 3b may still reflect a causal interpretation of surprise and competence. Second, another possibility is that some children may indeed have relied on simple associations to pass our task. However, the significantly diminished effect in Experiment 3b suggests that association-based explanations alone cannot fully account for our findings across all experiments.

The current results raise questions about the representations and inferential processes that underlie children’s judgments; how do children make the inferential leap from surprise to competence? One relatively lean explanation is that children in our study perceived surprise as a signal that the observed event is improbable. From this view, the inference does not rely on understanding others’ mental states; instead children only need to recognize that a surprised reaction to failure indicates the student was likely to do better, whereas a surprised reaction to success indicates the student was likely to do worse. Another possibility, however, is that children interpreted surprise as a signal that the observed event violated the emoter’s (i.e., the teacher’s) beliefs or expectations about the student. From this view, a surprised reaction to failure means the teacher *expected* the student to do better, whereas a surprised reaction to success means the teacher *expected* the student to do worse. Both accounts assume that children use surprise to draw inferences about competence, and teasing apart these possibilities is beyond the scope of the current work. However, given prior work suggesting that children, by age 5, have a causal understanding of how surprise reflects the emoter’s beliefs and expectations (Hadwin & Perner, 1991; MacLaren & Olson, 1993; Wellman & Woolley, 1990), it is likely that children who showed successful competence inferences in our study are at least capable of reasoning about competence via teacher’s beliefs. Future work might further test this hypothesis by asking whether children’s surprise-based inferences are mediated by the emoter’s beliefs.

The current study found successful inference about competence from surprise in early school-aged children (6- to 8-year-olds), but not younger children (4- and 5-year-olds). Yet, prior work, as reviewed earlier, has found that younger children show at least some understanding of others’ surprise. Even infants can use others’ surprised expressions to infer hidden properties of physical objects (Wu et al., 2024), and at least by age 5, they can connect others’ surprise with their knowledge or beliefs to draw inferences about the physical states of the world (Moll et al., 2006; Wellman & Banerjee, 1991; Wu & Gweon, 2021). What, then, explains the relatively late success in our study? We can consider at least two possibilities. First, what is inferred in our study—competence—is arguably more abstract than the target of inference in other studies, such as concrete objects or states of the physical world (e.g., where an object is or how a toy works; Hadwin & Perner, 1991; MacLaren & Olson, 1993; Moll et al., 2006; Wellman & Banerjee, 1991; Wu & Gweon, 2021). Consistent with prior work on children’s difficulty with grasping the concept of competence (Nicholls, 1978), using emotion to infer such an unobservable, internal quality of agents may be challenging for young children. Second, given that the age of success coincides with the start of formal schooling, it is also possible that experiences and social interactions in a school environment may prompt children to begin inferring competence from social cues (Magid & Schulz, 2015; Ruble et al., 1980), including emotional expressions. These factors are not mutually exclusive and may jointly contribute to the developmental change in our current work.

The following limitations leave open a number of questions and directions for future work. First, the current study used a binary, forced-choice measure by asking children to select between two students (i.e., “Who is better?”). While this measure is robust against task demands and can elicit reliable responses even from preschool-aged children, it is relatively coarse compared to more graded measures (e.g., ratings for each student) or open-ended verbal responses. Given that task demands can be confounded with genuine developmental change, in this study we made a deliberate design decision to focus on judgments of relative competence in a forced-choice paradigm. Yet, given the limitation of this measure, the use of other finer-grained measures or open-ended verbal responses may be useful for future work that build on these initial findings.

Second, the current study presented information about the teacher’s emotional reaction in two different ways: In addition to seeing the teacher’s facial expression on the screen, children were also verbally prompted to judge if the teacher was “surprised” or “not surprised”. While these prompts served primarily as comprehension checks, ensuring that children followed along and recognized the target emotions, they might have provided additional support to participants’ competence judgments by explicitly labeling the emotions. Granted, there are reasons to believe that facial expressions alone may be sufficient to drive children’s inferences. For instance, even preverbal infants can use others’ facial expressions of surprise to draw inferences about unobservable states of the physical world (Moll et al., 2006; Wu et al., 2024), and by around 5, children can spontaneously generate an appropriate verbal label for prototypical facial expressions of basic emotions, including surprise (Widen, 2013; Widen & Russell, 2003). Nonetheless, future research is needed to disentangle the respective contributions of facial expressions and linguistic labels, examining whether perceptual emotional cues alone are sufficient to drive children’s competence judgments.

Third, as the majority of our participants were from the United States, it is possible that the pattern of results might vary depending on sociocultural factors. Although the development of underlying representational and inferential capacities may be relatively comparable across cultures, there may be substantial variation in expectations about people’s reactions to others’ successes or failures or assumptions about what a teacher knows about a student. We look forward to future work using a range of scenarios and participant groups to examine the generalizability of these findings.

Lastly, our findings also highlight a broader direction for future work. Although the current study focused on 3rd-party judgments of relative competence based on emotional reactions towards two hypothetical characters (two students), in real-world contexts, such emotional reactions can be directed towards a range of targets to elicit powerful inferences with lasting social consequences. For instance, people might be particularly sensitive to emotional reactions that are directed at themselves, such as when a colleague or a teacher is surprised at their success. Recent work suggests that children as young as 3 years of age are sensitive to what others think of their abilities (Asaba & Gweon, 2022). Thus, it is possible that we might find similar, or even earlier, ability to draw emotion-based inferences when these reactions concern children’s own performance or qualities, such as their competence.

Furthermore, emotional reactions may support inferences about social groups when the target is perceived as a member of a social group. Research suggests that by age 6, girls are already less likely to believe that girls are smart and avoid activities that are described as being for children who are really smart (Bian et al., 2017). Compared to the role of generic linguistic cues in promoting and perpetuating stereotypes (e.g., Chestnut & Markman, 2018; Rhodes et al., 2012), relatively less is known about the role of emotional cues in the early emergence of such stereotypes. Even in supportive environments where adults avoid explicit, verbal remarks that imply gender stereotypes, it is possible that any underlying biases may manifest as emotional reactions. Although some prior work has suggested that children and adults can “catch” others’ social biases from observing their positive or negative reactions (Skinner et al., 2017, 2020), it’s possible that even subtle, non-valenced signals such as surprise may communicate biases (e.g., showing surprise when a girl does well in math). We hope that our findings will fuel future work on how stereotypes might emerge even from seemingly harmless, non-valenced emotional reactions to people.

Emotional cues may be fleeting, but they are powerful sources of information (Wu et al., 2021). By showing that reactions to everyday activities can elicit robust inferences about abstract, internal qualities of people even in young children, our study opens up new lines of inquiry about their underlying mechanisms as well as ways to minimize their unintended consequences.

## ACKNOWLEDGMENTS

We thank Stephanie Chang for help with data collection, Kate Littlejohn for help with coding, and the Palo Alto Junior Museum & Zoo, and families who participated.

## FUNDING INFORMATION

This work was supported by an NSF Graduate Research Fellowship to M.A. (Stanford University); the Paul & Lilah Newton Brain Science Award to Y.W.; a McDonnell Scholars Award from the James. S. McDonnell Foundation, a Jacobs Foundation Research Fellowship, and an NSF award (BCS-2019567) to H.G.

## AUTHOR CONTRIBUTIONS

M.A.: Conceptualization; Formal analysis; Investigation; Methodology; Project administration; Validation; Visualization; Writing – original draft; Writing – review & editing. Y.W.: Conceptualization; Formal analysis; Investigation; Methodology; Project administration; Validation; Visualization; Writing – original draft; Writing – review & editing. B.C.: Investigation, Methodology, Writing – review & editing. H.G.: Conceptualization, Funding acquisition, Methodology, Resources, Supervision, Writing – original draft, Writing – review & editing.

## DATA AVAILABILITY STATEMENT

Preregistrations, materials and experimenter scripts, data, and analyses are here: https://osf.io/4ezvt/.

## Notes

^1^ Technically, one’s surprised reaction can only inform inferences about their beliefs (i.e., prior expectations) rather than the actual ground truth. As an initial step, the current work focuses on cases where the observer’s beliefs are likely to be accurate, such as a teacher’s beliefs about a student’s competence.^2^ Our key manipulation was the presence or absence of surprise. In order to keep this manipulation consistent across both failure and success conditions, we deliberately used a mildly positive expression (i.e., a faint smile) as both the baseline and the no-surprise response. A truly neutral expression is often perceived negatively in social contexts (Chiarella & Poulin-Dubois, 2015), making it unsuitable for the baseline expression and especially unsuitable as a response to success. While other responses (e.g., empathy) may be deemed more plausible or socially appropriate for failure, such responses are difficult to convey using facial expressions, and cannot be used in success scenarios. Given that a mildly positive expression could also signal support and positive encouragement in response to failure, we opted to use it both as a baseline and as the expression to indicate an absence of surprise.^3^ The kicking and math activities were always shown in the same condition.

## Supplementary Material


