# Supplementary material for: When Success Is Surprising: Children’s Ability to Use Surprise to Infer Competence
Source: Open Mind (Camb). 2025 Jul 7;9:825–43. doi: 10.1162/opmi.a.2 (PMC12283150; doi:10.1162/opmi.a.2)
Supplement: Supplementary file 1 [file opmi-09-825-s001.pdf]

Supplementary Online Materials

Supplementary materials for "When success is surprising: Children's ability to use surprise to infer competence" contain reports of a replication of Experiment 1, pilot study for Experiment 2, and supplementary analyses for all experiments, and a supplemental methods figure.

1 Replication of Experiment 1

1.1 Methods

1.1.1 Participants

Sixty-seven adults (M_{Age}(SD) = 36.9(10.9), range = 19-64, 39 female, 28 male) were recruited from *Amazon's Mechanical Turk*. An additional 4 subjects were excluded for failing 50% (8 of 16) or more of the post-test check questions.[1]

1.1.2 Procedure

As in Experiment 1, participants viewed an online survey on *Qualtrics*. The procedure was similar to Experiment 1, except that participants underwent 8 trials total (4 success trials, 4 fail trials), rather than only 4. Each trial showed two same-gender students who both succeeded (success condition) or failed (fail condition) at the same activity. The key difference between the two students was whether the teacher expressed surprise (henceforth "surprise student") or did not express surprise ("no-surprise student") at the student's outcome. Participants responded to a test question ("Who is better at math?"), followed by two post-test check questions ("Just to check, was the teacher surprised or not surprised that John/Noah made the ball into the goal?").

1.2 Results and Discussion

Our primary question was whether adults would use the teacher's surprised (versus non-surprised) expression to the students' performance outcomes to infer their relative competence.

[1] Including these participants does not qualitatively change the results of the study.

First, using the same analytical plan as in Experiment 1, we ran a generalized linear mixed-effects model with Condition (0 = fail, 1 = success) as a fixed effect predicting participants' choice for the student who received surprise; we included random intercepts for Activity and random intercepts and slopes for Participants by condition. We find a strong main effect of Condition ($\beta = -7.939$, $z = -6.386$, $p < .001$), suggesting that participants chose different students across conditions. Second, consistent with our predictions, participants selectively chose the no-surprise student in the Success condition (92.2%, $Z = 7.45$, $p < .001$) and the surprise student in the Fail condition (93.0%, $Z = 7.51$, $p < .001$, Exact Wilcoxon-Pratt Signed-Rank Test).

These results replicate our findings from Experiment 1, and provide corroborating support that emotional responses inform people's inferences about relative competence. When two agents both succeeded at the same task, the agent to whom the teacher expressed surprise was judged as less competent. In contrast, when both failed, the agent to whom the teacher expressed surprise was judged as more competent. These findings suggest that participants integrate surprised expressions (the teacher's reaction to an outcome) with external events (e.g., how the students performed) to infer others' relative competence.

2 Pilot for Experiment 2

Before conducting Experiment 2 (in the main text), we first ran an in-person pilot study with a relatively broad age range of children (4- to 9-year-olds). The purpose of this pilot was to determine the age range for our full, preregistered sample for Experiment 2.

2.1 Methods

2.1.1 Participants

Twenty-eight children (M_{Age}(SD) = 6.6(1.7), range = 4.1-9.9, 13 girls, 15 boys) were recruited from a local museum (n = 20) and campus preschool (n = 8). We excluded one 4-year-old participant who failed 50% or more of the check questions, and one 6-year-old who responded with "both" to

all questions (predetermined exclusion criteria).

2.1.2 Stimuli

Participants were shown images of the teacher's surprised and non-surprised expressions on laminated paper for warm-up questions at the beginning of the experiment. For the test trials, children were shown the same images from Experiment 2 on laminated paper.

2.2 Procedure

The procedure was similar to Experiment 2, except participants underwent 8 trials (similar to the adult replication experiment above), instead of 4 trials. Participants first underwent the warm-up task, where they were asked to identify the teacher's emotional expressions ("Is the teacher surprised or not surprised here?"). Then, they underwent 8 test trials that were structured the same as in Experiment 2: the experimenter first introduced one student, their performance outcome, and their teacher's emotional reaction to the outcome (e.g., surprise); then the experimenter introduced the other student, their identical outcome, and their teacher's other emotional reaction (e.g., no surprise). Participants were asked to choose which student is better at the activity; they responded by pointing to one of the students or verbally responding with their name. Fail and Success trials were paired and randomized within activity; activity order was randomized. Each participant underwent all 8 trials.

2.3 Results

All participants correctly identified the teacher's emotional expressions ("surprised" or "not surprised") in the warm-up questions. For the check questions in each trial about the teacher's emotional response to the student's performance ("surprised" or "not surprised"), the majority of participants (25 of 28) correctly answered all 16 questions; the remaining correctly answered 10 (n = 1 4-year-old), 14 (n = 1 5-year-old), and 15 (n = 1 9-year-old) of the questions.

We ran a generalized linear mixed-effects model with Condition (fail, success) and Age (continuous) as fixed effects, with an interaction term between Condition and Age, predicting participants' choice of student; we included random intercepts for Activity and random intercepts and slopes for Participants by Condition. We find main effects of Condition ($\beta = -3.258$, $z = -4.443$, $p < .001$), Age ($\beta = 1.006$, $z = 2.618$, $p = .009$), and a significant interaction between the two ($\beta = -2.711$, $z = -5.238$, $p < .001$).

As a group, children chose the no-surprise student in the Success condition (71.4%, $Z = 2.91$, $p = .004$, Exact Wilcoxon-Pratt Signed-Rank Test). In the Fail Condition, their choices were in the predicted direction but did not reach significance (59.8%, $Z = 1.32$, $p = .211$). Although it appears that there is an asymmetry in children's performance between the Success and Fail conditions (better performance in the Success condition), we observed the opposite asymmetry in Experiment 2 and did not observe an asymmetry in Experiment 3a. Thus, it appears that the asymmetry observed in this experiment is not robust.

Given the significant Age-by-Condition interaction and the relatively wide age range, we median-split children into younger (age range: 4.1 - 5.9; n = 14) and older age groups (age range: 6.2 - 9.9; n =14) and looked at children's choices within each trial. We found that the older children (6-9 year-olds) showed above-chance performance in both the Success and Fail conditions (Success: 98.2% choice for the no-surprise student, $Z = 3.64$, $p < .001$; Fail: 76.8% choice for the surprise student, $Z = 2.16$, $p = .039$). The younger children (4-5 year-olds) were at chance for both trial types (Success: 44.6% choice for the no-surprise student, $Z = -.58$, $p = .707$; Fail: 42.9% choice for the surprise student, $Z = -.81$, $p = .536$).

Collectively, these results provided initial evidence for children's developing abilities to judge others' relative competence from emotional responses to performance outcomes. Given that these abilities were observed only in the older participants (6-9 year-olds) but not the younger ones (4-5 year-olds), we decided to run a full, preregistered sample with 6-8 year-olds (Experiment 2 in the main text).

3 Supplementary Analyses

3.1 Experiment 1

3.1.1 Individual-level performance

We explored how many individual participants showed the predicted pattern for 1, 2, 3, or 4 (all) trials. We found that 83.3% of participants (50 of 60) responded with the predicted pattern to all 4 trials, while 8.33% (5 of 60) responded with the predicted pattern to 3 trials and 8.33% (5 of 60) responded to 2 trials.

3.2 Experiment 2

3.2.1 Explanation of pre-registered model

We pre-registered a model that neither converged nor was ideal. The model was a mixed-effects logistics regression with Condition (Success, Fail), Activity (throwing, kicking, math, spelling), Age (continuous), Order (first or second block of trials), and an interaction term between Condition and Age as fixed effects, and Participant as a random intercept, predicting which student participants chose as better: glmer(Choice Condition + Age + Order + Activity + Condition:Age + (1—Participant)). The model failed to converge with max—grad— = .4427. Besides the convergence issue, we also recognized, after counselling a statistics expert, that we should run a maximal model with Activity as a random effect rather than a fixed effect (**?**). We opted to use the model suggested by the expert in both of our experiments, as reported in the main paper.

3.2.2 Individual-level performance

We observed that 52.22% (47 of 90) responded correctly to all 4 trials, 8.88% (8 of 90) responded correctly to 3 trials, 33.33% (30 of 90) responded correctly to 2 trials, 3.33% (3 of 90) responded correctly to 1 trial, and 2.22% (2 of 90) participants responded correctly to no trials.

3.3 Experiment 3a

3.3.1 Mixed-effects model for 6- to 8-year-olds

To enable comparisons with Experiment 2, we ran exploratory analyses focusing on 6- to 8-year-olds alone (matching the same age range as in Experiment 2). We used the same mixed-effects logistic regression as the preregistered analysis reported in Experiment 3a. As the full model failed to converge, we pruned the random-effect structure following the preregistered pruning procedure for the main analysis, and the model converged after removing the Condition random slope for participants. The results are reported in the main text.

3.3.2 Individual-level performance

For age groups that showed the expected condition difference (i.e., chose different students between the Success and Fail conditions as shown by the regressions: 6-, 7-, and 8-year-olds), we explored performance at the individual level. We found that 45.56% (41 of 90) participants responded correctly to all 4 trials, 13.33% (12 of 90) responded correctly to 3 trials, 31.11% (28 of 90) responded correctly to 2 trials, and 10% (9 of 90) responded correctly to 1 trial.

3.4 Experiment 3b

3.4.1 Individual-level performance

The main text reports the % of partcicipants who showed the "predicted" pattern (from Experiment 3b) on all 4 trials. In addition, 16.7% (15 of 90) showed the "predicted" pattern in 3 trials, and a majority of participants showed this pattern in 2 trials (53.3%, 48 of 90) or less (12.2%, 11 of 90 in one trial, 3.3% (3 of 90) to none).

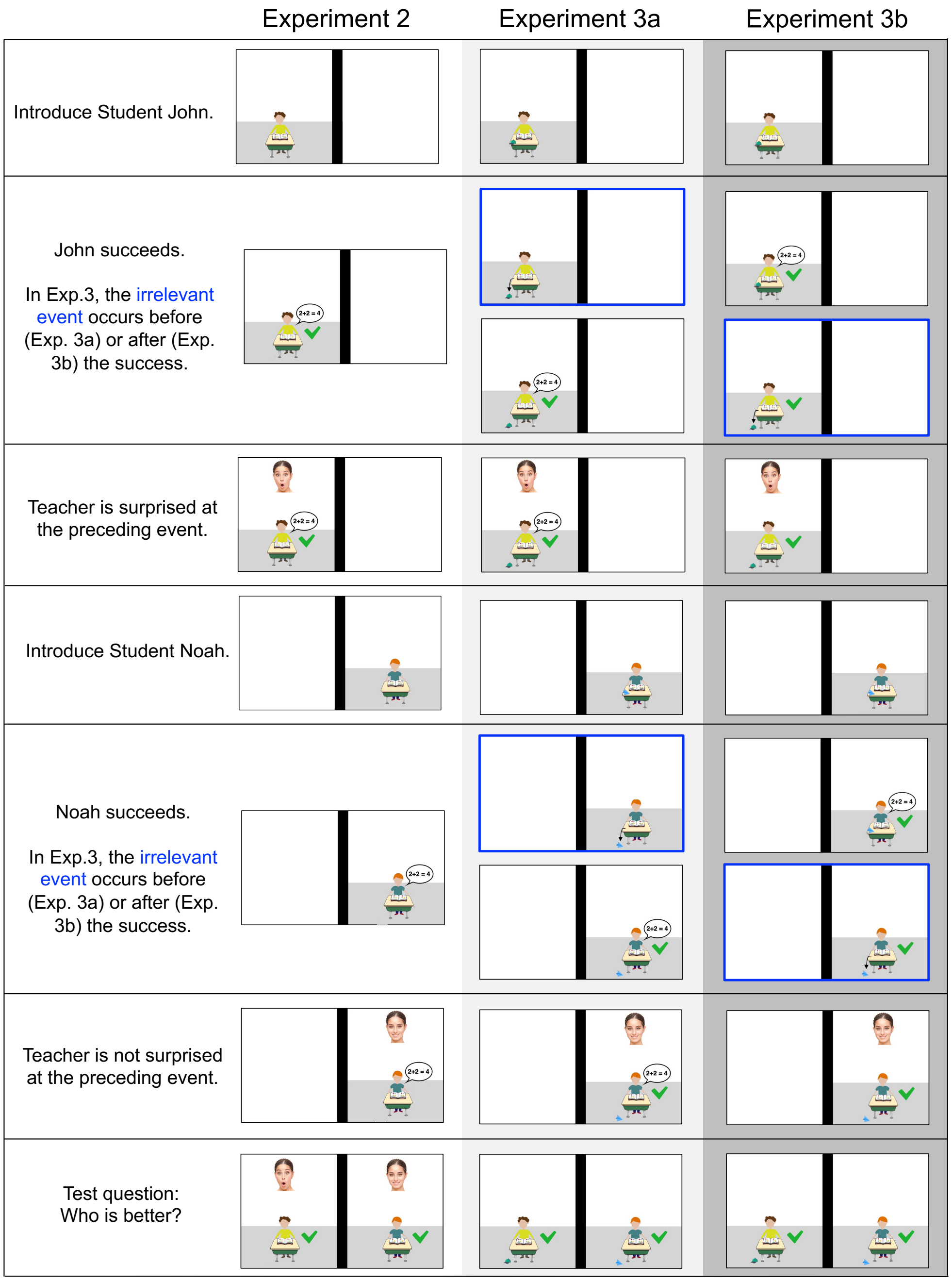

Figure S1: Procedure for the math trial in the Success Condition in Experiments 2, 3a, and 3b (control). The procedure was similar across experiments: Participants saw two students, one at a time, who each succeeded on the same task (e.g., a math problem). In Experiments 3a and 3b, there was an additional, irrelevant event (in blue) that occurred before (Experiment 3a) or after (Experiment 3b) the student's success. Then, the teacher showed surprise or no surprise to the preceding event, which was either the student's success (Experiments 2 and 3a) or the irrelevant event (Experiment 3b). The Fail condition was identical, except both students failed at the task. Note that in Experiment 1, the stimuli were similar, but the procedure was condensed such that both students were shown simultaneously.
